# The first mitochondrial genome of the living-fossil sawfly *Macroxyela ferruginea* (Hymenoptera: Xyelidae, Macroxyelinae)

**DOI:** 10.1080/23802359.2019.1694849

**Published:** 2019-12-09

**Authors:** Bo-Ying Zheng, Ze-Kai Li, Xiao-Fei Li, Jia-Chen Zhu, Michael Sharkey, Pu Tang, Xue-Xin Chen

**Affiliations:** a Ministry of Agriculture Key Lab of Molecular Biology of Crop Pathogens and Insects, Zhejiang University, Hangzhou, China;; b Institute of Insect Sciences, Zhejiang University, Hangzhou, China;; c Zhejiang Provincial Key Lab of Biology of Crop Pathogens and Insects, Zhejiang University, Hangzhou, China;; d State Key Laboratory of Rice Biology, Zhejiang University, Hangzhou, China;; e Department of Entomology, University of Kentucky, Lexington, KY, USA

**Keywords:** Mitochondrial genome, living-fossil sawfly, Xyelidae; *Macroxyela*, phylogeny

## Abstract

The living-fossil sawfly *Macroxyela ferruginea* (Xyelidae: Macroxyelinae) was one of the oldest species of Hymenoptera. We sequenced the mitochondrial genome, 15,465 bp in size. All 37 typical mitochondrial genes were possessed. There is only one rearrangement of gene order, where *trnM* and *trnQ* were shuffled. We also found this order was shared with *Xyela* sp., which also belongs to family Xyelidae. The 13 protein-coding genes of this sequence and the other 10 species from eight superfamilies in Hymenoptera were all used for phylogenetic analysis by maximum likelihood (ML) analysis and Bayesian inference (BI), with *Ascaloptynx appendiculatus* from Neuroptera as an outgroup. The topology demonstrated that *M. ferruginea* was sister to *Xyela* sp., supporting that they belong to one family Xyelidae.

The living-fossil sawfly *Macroxyela ferruginea* belongs to the genus *Macroxyela* within the subfamily Macroxyelinae (Hymenoptera: Xyelidae) (Smith and Schiff 1998). The superfamily Xyeloidea, a poor-species lineage, only has one family Xyelidae and two extant subfamilies, Macroxyelinae and Xyelinae (Taeger et al. 2010). Furthermore, Xyelidae, with numerous fossil records, is sister to all other extant Hymenoptera. *Macroxyela* is only distributed in North America (Smith and Schiff 1998). Its closest lineage is the genus *Megaxyela* which occurs only in eastern Asia and eastern North American (Blank et al. 2017). Typically, *Macroxyela* is rarely collected, although *M. ferruginea* can be often found on its host angiospermous trees (Smith and Schiff 1998). In terms of morphology, its long hind legs and colorful body are worthy of note.

In this study, we got the mt-genome of *M. ferruginea* by next-generation sequencing. The sample was collected from Plummers Island (Cabin John, MD, USA) (38°58′10″N, 77°10′35″W) during March 1999. After sampling, the voucher specimen (ZJUH_190001) was stored in 100% ethanol and kept in the Parasitic Hymenoptera Collection of Institute of Insect Sciences, Zhejiang University. The whole genomic DNA was extracted from one female adult specimen using DNeasy tissue kit (Qiagen, Hilden, Germany) and remaining specimen is deposited there. The library was constructed by VAHTS^TM^ Universal DNA Library Prep Kit for Illumina^®^ v3, and sequenced by Illumina HiSeq X Ten sequencer (150 bp pared-end). The reads were filtered by local BLAST with *E* value 1 × 10^−5^ referencing to all Symphyta mitochondrial genomes dataset, subsequently, assembled by IDBA_UD (Peng et al. 2012).

The mitochondrial genome of *M. ferruginea* is 15,465 bp in length (GenBank accession MK270536), containing 13 protein-coding genes (PCGs), 22 transfer RNA (tRNA) genes, and two ribosomal RNA (rRNA) genes, but part of the control region (D-loop) failed to be sequenced or assembled. All genes show the conservative arrangement compared with the ancestral type of *Drosophila melanogaster*. The only rearrangement of gene order is that *trnM* and *trnQ* were shuffled in this mitogenome sequence. In addition, we compared the order of genes of *M. ferruginea* with its closest lineage *Xyela* sp. from another subfamily Xyelinae (Tang et al. 2019). Both have the same arrangements. The same gene order shared by different subfamilies in mitogenomes evidenced the conservative evolution of this group compared to the other lineages of Hymenoptera.

The complete or nearly complete mitochondrial genomes of the family Xyelidae is very limited, with only one reported species *Xyela* sp. The 13 PCG sequences of *M. ferruginea* and other 10 species from eight representative superfamilies were used for phylogenetic analysis by maximum likelihood (ML) methods in software IQtree v1.6.12 (Nguyen et al. 2015) and Bayesian inference (BI) in MrBayes v3.2.549 (Ronquist et al. 2012), with *Ascaloptynx appendiculatus* from Neuroptera as an outgroup. *M. ferruginea* is sister to *Xyela* sp., a species also belonging to Xyelidae, with a confidence value (bootstraps = 100, posterior probability = 1), a basal group to other lineages of Hymenoptera (Figure 1). This result was consistent with other results from the analyses based on morphological characters and several sequences before (Schulmeister 2003; Sharkey et al. 2012; Ma et al. 2019).

**Figure 1.**
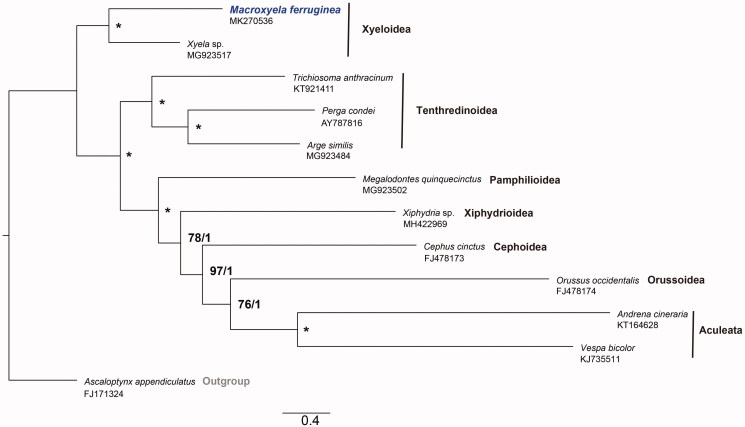
Phylogenetic tree of maximum likelihood (ML) methods and Bayesian inference (BI) using matrixes of 13 PCGs in mitochondrial genomes of 11 representative lineages in Hymenoptera and one outgroup *Ascaloptynx appendiculatus*. The support values of the corresponding nodes are shown above (left is bootstraps of ML, right is posterior probability of BI, “*” indicates that bootstraps = 100 and posterior probability = 1).
